# The Relationship between Smoking, Socioeconomic Status and Grip Strength among Community-dwelling Elderly Men in Korea: Hallym Aging Study

**DOI:** 10.4178/epih/e2013001

**Published:** 2013-02-18

**Authors:** ShanAi Quan, Jin-Young Jeong, Dong-Hyun Kim

**Affiliations:** 1 Department of Social and Preventive Medicine, Hallym University College of Medicine, Chuncheon, Korea.; 2 Hallym Research Institute of Clinical Epidermiology, Hallym University College of Medicine, Chuncheon, Korea.; 3 Korea Health Promotion Foundation, Seoul, Korea.

**Keywords:** Elderly men, Hand strength, Smoking, Socioeconomic

## Abstract

**OBJECTIVES:**

Low grip strength is associated with decline in bone mineral density (BMD) and increased risk of spine fracture among the elderly. Smoking, a major factor determining BMD, is also known to have an indirect effect on bone loss. This study investigated whether smoking is associated with grip strength in the community-dwelling elderly in Korea.

**METHODS:**

This study was an outcome of the second of three waves of the Hallym Aging Study from January to May 2007, a population-based study of Koreans aged 45 years and upwards dwelling in Chuncheon. Its 218 subjects comprised men aged 65 years or over. They were evaluated at a general hospital for socioeconomic status, smoking history, and various clinical measures including grip strength.

**RESULTS:**

Grip strength was higher in non-, ex-, and current smokers (33.7 kg, 30.6 kg, and 29.3 kg, respectively). Current smoking was found to increase the risk of decreased grip strength (adjusted odds ratio [aOR], 4.58; 95% confidence interval [CI],1.31 to 16.04) compared with non-smoking, after adjustment for potential covariates including socioeconomic status. After adjustment for smoking effect, education of fewer than six years and monthly income of fewer than 500,000 Korean won increased the risk of decreased grip strength compared with education of more than six years (aOR, 2.88; 95% CI, 1.08 to 7.66) and monthly income of more than 1,500,000 Korean won (aOR, 2.86; 95% CI, 1.08 to 7.54).

**CONCLUSIONS:**

These results showed that current smoking, low education and low income were independent risk factors for decreased grip strength among elderly men in Korea.

## INTRODUCTION

Physical functioning represents an integrated marker of aging, influenced by a broad array of physiological and clinical characteristics interacting with behavior and the social environment [1]. In old age, decreased muscle strength predisposes people to functional limitations and disability [2]. Older people had less appendicular skeletal muscle than younger, the decline in muscle mass of -0.4 kg/decade in women and 0.8 kg/decade in men starting at the age of 20 [3]. According to Rantanen et al. [4], the intra-individual strength changes over time were significant in all ages. Muscle strength was found to influence life-span. Those who have higher grip strength during midlife remain stronger than others in old age [4] and people who have greater muscle strength during midlife then have a lower risk of becoming disabled because of their greater ability to maintain that strength regardless of chronic conditions that may develop [5]. Grip strength is known as a good predictor of unhealthy outcomes such as mortality and may indicate subclinical disease, which later develops into clinical disease [5] and functional limitation [6]. Grip strength is also known to be correlated with bone mineral density (BMD) in middle-aged men [7]. Age-related bone loss is reported to be associated with decreased BMD [8] and with weakened muscle strength [9]. Grip strength tests are convenient, safe, and reliable, and do not require large or expensive equipment [10]. Consequently, grip strength has been used as an indicator of overall muscle strength in many population studies [11].

Smoking is one of the major components determining BMD [12]. Smoking is associated with lower BMD of neck and spine in both male and female elderly and is known to increase hip fracture rates compared with nonsmokers [10]. In Ward and Klesges [11] study, absolute effects at most bone sites were greatest for current smokers compared with those who had never smoked.

Elderly with lower socioeconomic status (SES) according to educational attainment, income, and occupation have significantly higher morbidity and mortality than individuals with higher SES [13,14]. A study has shown that SES is related to mobility disability in later life [15]. Levels of education and/or income have also been significantly associated with decreased physical function [14,16]. Studies have found that higher SES is associated with higher BMD at total hip [17], femoral neck [18], and spine [19].

This study was conducted to elucidate whether smoking and social economic status are associated with grip strength in community-dwelling elderly men in Korea.

## MATERIALS AND METHODS

### Subjects

**Figure 1:**
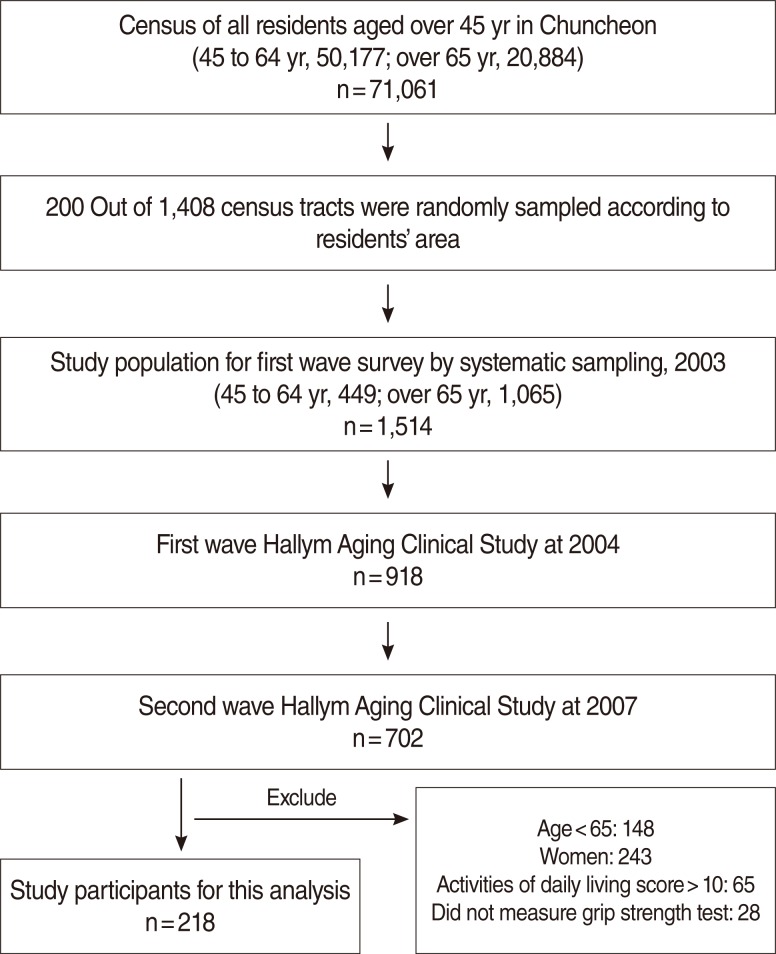
Flow chart of Hallym Aging Study.

Subjects in this analysis were recruited in the Hallym Aging Study (HAS). HAS is a population-based study of Koreans aged 45 years or older dwelling in Chuncheon. The first wave began in 2003 and an in-depth clinical study was started in 2004. The city was divided into 1,408 areas based on the 2000 census and 200 were randomly selected. The first-wave participants were selected by systematic sampling: 30% of subjects were sampled from individuals aged 45 to 64 and 70% were sampled from individuals aged 65 years or older. Participants of the first-wave survey in 2003 numbered 1,520. Among them, 918 had participated in an in-depth clinical study in 2004. A physical performance test was administered in the second wave of the clinical study in 2007, whereby the subjects were limited to elderly men aged 65 years or older, excluding 93 individuals who did not test for hand grip or had a score of activities of daily living score>10. Finally, 218 subjects were available for the final analysis (Figure 1).

Demographic characteristics included age, marital status, average monthly income and education. Age was divided into 65 to 69, 70 to 74, 75 to 79, and 80 years and older groups. Monthly income was divided into fewer than 500,000, 500,000 to 1,490,000, 1,500,000 to 2,990,000 and more than 3,000,000 Korean won (1,000 Korean won is about 1.00 US dollar). Education level was divided into 0 to 6, 6, and more than 6 years. Smoking status was categorized as current smoker, past smoker, and never.

### Test of grip strength

To test grip strength, each participant sat on a chair and put one arm on the table in front of him. The wrist and forearm were in neutral anatomical position and the arm was flexed at 90°. In this position, the participants were encouraged to exert their maximal grip [20]. Grip strength was measured for both hands and every hand measured twice. We checked the main hand, let participants rest for 15 seconds before the second test, then swapped hands. Grip strength measurement was performed with the digital dynamometer (TKK-5401; Takei, Tokyo, Japan) in kilograms, and the better numerical value of the main hand was used in the analysis.

### Statistical analysis

Characteristics of study participants according to grip strength were expressed by mean with standard deviation for continuous variables and by number with percentages for categorical variables. Least-squares mean for grip strength according to smoking status was calculated by the PROC GLM program (SAS Inc., Cary, NC, USA). Since some factors, such as age, lifestyle-related factors, genetic background, physical activities, and previous medical diseases, may affect grip strength decline [21], odds ratios (OR) for risk of decline of grip strength were computed by logistic regression analysis after adjustment for potential covariates. Grip strength was divided into low and high groups by median. Data analyses were performed with the SAS version 9.1 (SAS Inc., Cary, NC, USA).

## RESULTS

**Figure 2:**
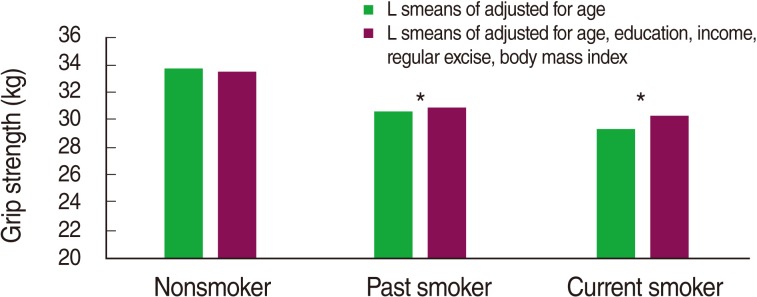
Grip strength according to smoking status among men. ^*^p<0.05 compared to nonsmokers.

Table 1 shows overall characteristics of study subjects according to level of grip strength. The subjects with high grip strength were younger, more educated, more likely to be smokers, and more likely to participate in regular exercise than those with low grip strength (Table 1). The grip strength of nonsmokers adjusted for age, smoking status, level of education, and obesity was higher than that of past and current smokers (Figure 2).

After adjustment for age, education, income, regular excise, and body mass index, the risk of decreased grip strength gradually increased among current smokers (aOR, 4.5; 95% CI, 1.10 to 9.17), those with low educational attainment (aOR, 2.88; 95% CI, 1.08 to 7.66) and those with low monthly income (aOR, 2.86; 95% CI, 1.08 to 7.54), compared with non-smokers, those with high educational attainment, and those with high monthly income, respectively (Table 2).

## DISCUSSION

Current smokers were found to have higher risk of decreased grip strength compared with non-smokers. Education and income also affected the risk of the decline of grip strength among the elderly men in this study.

Hands have many physiological and anatomical changes associated with aging. Impaired hand abilities are observed among the elderly with metabolic and skeletal diseases such as osteoarthritis and rheumatoid arthritis [22]. Starting in middle age, men lose approximately a fifth of BMD over their lifetimes [23]. Smokers were reported to have less bone mass at all measured sites, and smoking cessation had a beneficial effect on bone mass as past smokers had an intermediate bone phenotype compared with either lifelong non-smokers or current smokers [24]. Smoking is significantly associated with BMD in middle-aged men [25,26] since the nicotine from cigarettes is involved in the metabolism of calcium and vitamin D and affects bone health [27]. Smokers may have different taste perception, leading them to choose unhealthy food [28], and may take less exercise [29] compared with non-smokers. Kanis et al. [30] reported that current smoking was associated with a 25% increase in fracture risk compared with the risk in subjects who had never smoked. One study of elderly men and women reported grip strength can predict BMD of the proximal radius [31] and another study of athletes also reported that grip strength was a predictor of radial BMD [32].

Low income and low educational attainment can affect the risk of overall health and functional limitations among the elderly [33] and are known to be associated with lower levels of self-efficacy in specific behavioral domains [34]. High income and educational attainment are more likely to have a higher level of perceived control and thus situation-specific self-efficacy [35]. This means that higher socioeconomic status may increase the likelihood of individuals having the confidence necessary necessary to attempt socially prescribed behavioral changes and having the resources necessary to ease the adoption process (e.g., time, money) [33]. Higher education may allow someone to access more knowledge on health, practice more healthy behaviors, have more employment opportunities, and earn higher income, which all affect health [36]. Income affects health in various ways, such as availability of material resources and health services [15].

Given the public health implications of smoking on bone health, it is important that this information be incorporated into smoking prevention and cessation efforts targeting the elderly [37]. Elderly people commonly have difficulties in terms of hand function and hand strength can affect simple everyday actions. Grip strength is a simple test and an important index of the body's muscle situation and can predict disability in the nearer future. Declining physical fitness as a natural aging process may not be completely prevented, however.

One of the strengths of this study is that the long-term effect of smoking on physical function and occurrence of chronic diseases can be assessed in a relatively short time through a test of grip strength, which can be regarded as a surrogate indicator predicting these health conditions. This study may not appropriate to demonstrate the effect of smoking on health outcomes directly since we observed them at the same time in this cross-sectional study.

A limitation of this study is that there may be residual confounding owing to categorical variables such as smoking (non-, ex-, and current) and socioeconomic status. It is hard to exclude the possibilities of unknown confounders between groups with high and low grip strength. In addition, because participants in this study at least maintained their health, it was not representative of all elderly people, but the findings can be applied to healthy elderly people residing in the community.

Though we cannot change some factors such as age and education among the elderly, we can improve health behaviors such as smoking, and reverse or delay the process of decline of muscle strength.

## Figures and Tables

**Table 1:**
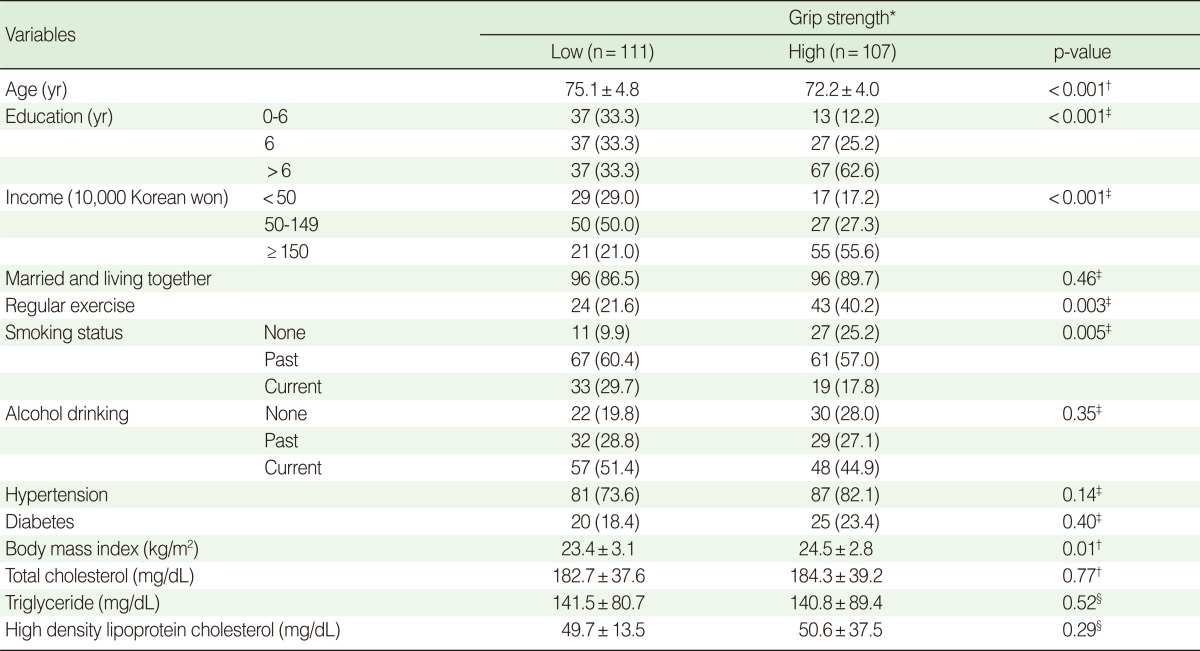
Characteristics according to grip strength at baseline

Values are presented as mean±SD or number (%). ^*^Cut point of grip strength for men is 31.4 kg; ^†^Two sample t-test; ^‡^Chi-squared test; ^§^Wilcoxon's rank sum test.

**Table 2:**
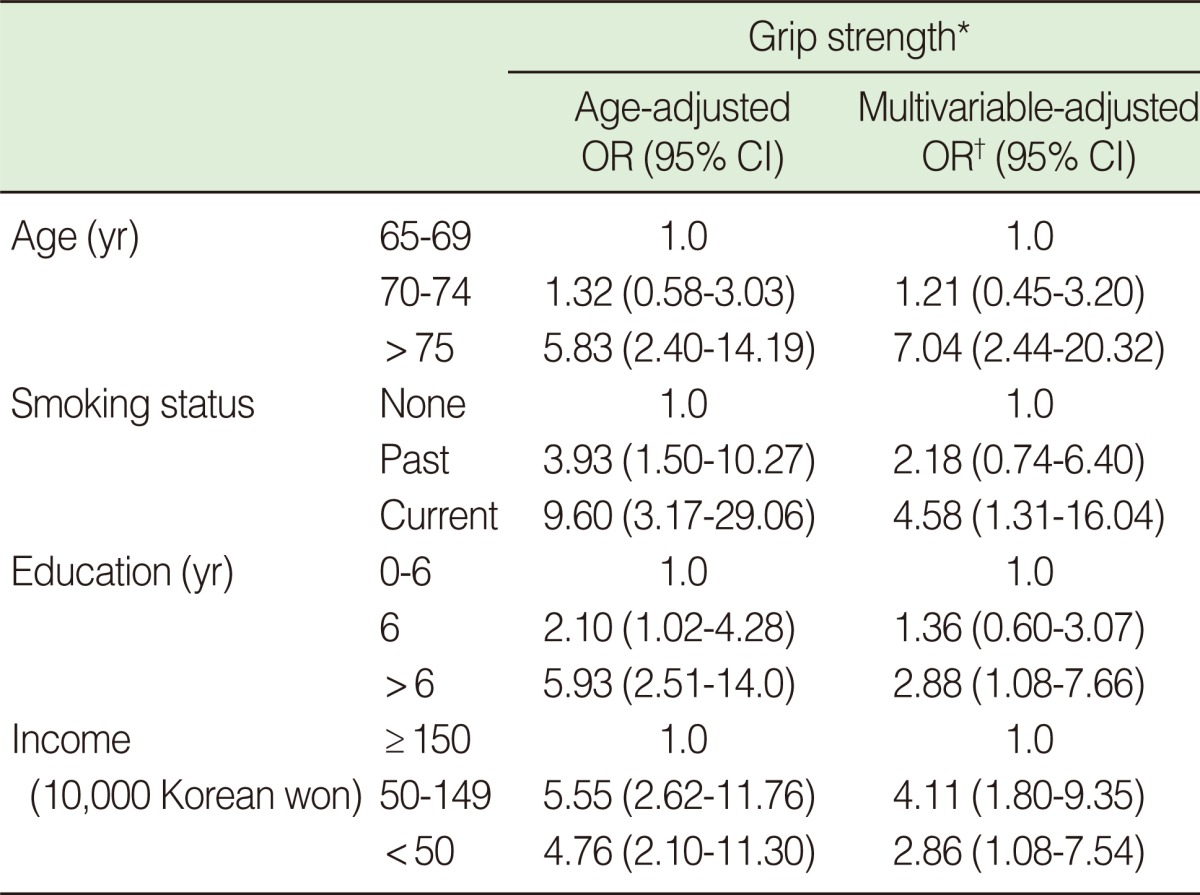
Effect of smoking status on the risk of low grip strength among elderly men

OR, odds ratio; CI, confidence interval. ^*^Cut point of grip strength for men is 31.4 kg; ^†^Adjusted for age, education, income, regular excise, body mass index smoking.
